# Full-waveform inversion based on generalized Rényi entropy using patched Green’s function techniques

**DOI:** 10.1371/journal.pone.0275416

**Published:** 2022-11-11

**Authors:** Wagner A. Barbosa, Sérgio Luiz E. F. da Silva, Erick de la Barra, João M. de Araújo

**Affiliations:** 1 Department of Theoretical and Experimental Physics, Federal University of Rio Grande do Norte, Natal, RN, Brazil; 2 Department of Applied Science and Technology, Politecnico di Torino, Torino, Italy; 3 Seismic Inversion and Imaging Group, Fluminense Federal University, Niterói, RJ, Brazil; 4 Department of Physics, University of La Serena, La Serena, CO, Chile; 5 Department of Mathematics, University of La Serena, La Serena, CO, Chile; Tongji University, CHINA

## Abstract

The estimation of physical parameters from data analyses is a crucial process for the description and modeling of many complex systems. Based on Rényi *α*-Gaussian distribution and patched Green’s function (PGF) techniques, we propose a robust framework for data inversion using a wave-equation based methodology named full-waveform inversion (FWI). From the assumption that the residual seismic data (the difference between the modeled and observed data) obeys the Rényi *α*-Gaussian probability distribution, we introduce an outlier-resistant criterion to deal with erratic measures in the FWI context, in which the classical FWI based on *l*_2_-norm is a particular case. The new misfit function arises from the probabilistic maximum-likelihood method associated with the *α*-Gaussian distribution. The PGF technique works on the forward modeling process by dividing the computational domain into outside target area and target area, where the wave equation is solved only once on the outside target (before FWI). During the FWI processing, Green’s functions related only to the target area are computed instead of the entire computational domain, saving computational efforts. We show the effectiveness of our proposed approach by considering two distinct realistic P-wave velocity models, in which the first one is inspired in the Kwanza Basin in Angola and the second in a region of great economic interest in the Brazilian pre-salt field. We call our proposal by the abbreviation *α*-PGF-FWI. The results reveal that the *α*-PGF-FWI is robust against additive Gaussian noise and non-Gaussian noise with outliers in the limit *α* → 2/3, being *α* the Rényi entropic index.

## Introduction

Full-waveform inversion (FWI) is a powerful methodology to estimate subsurface physical parameters by exploring the complete waveforms recorded in a seismic survey [1, 2]. From a practical point of view, FWI is formulated as a local optimization problem, in which the misfit function to be minimized is often based on the least-squares distance between the modeled and observed data [3]. In this regard, the modeled data are obtained by solving the wave equation in an area of the subsurface model, which is associated with the seismic acquisition geometry, in order to compare with the observed data. Indeed, the construction of quantitative models from the ample physics provided by the wave equation solution is very useful for describing and modeling complex systems. For this reason, this technique has been employed in a wide variety of applications from geophysics [4–6] to other scientific fields such as biomedical imaging [7, 8] and astrophysics [9, 10].

Despite the FWI potentials, it is inherently an ill-posed problem in the sense of Hadamard, which means that at least one of the following features is violated: the solution (i) exists, (ii) is unique; and (iii) depends continuously on the observed data. The characteristic (ii) is commonly violated in FWI because in a typical geophysical survey there is information only on the positions associated with the seismic receivers that cover a small area of the region of interest, which leads the FWI to solve an inconsistent and overdetermined system of equations. Regarding the characteristic (iii), the FWI solution may be unstable, as a small variation in the noise level of the observed data can lead to discontinuous changes in the reconstructed subsurface model [2]. Furthermore, the least-squares FWI (hereinafter classical FWI) assumes that the errors obeys Gaussian statistics [11], which is not always true, for instance, in geophysical problems [12, 13].

In the FWI case, the errors can come from the computation of differences (or other criteria such as similarity measures) between the modeled and observed data, and therefore, it includes uncertainties associated with seismic noise and incomplete modeling of wave physical phenomena. Indeed, errors are seldom Gaussian in FWI applications. Thus, a wide variety of criteria has been proposed in the literature to mitigate the effects of non-Gaussian errors in the data inversion process [14]. A very common robust criterion to non-Gaussian errors, especially to erratic data (outliers), is the misfit function based on the *l*_1_-norm of the error. Such criterion is based on the assumption that the errors obey the Laplace distribution. Its success is associated with the long tails of the Laplace distribution [12]. For this reason, inverse problems based on the Laplace distribution have been extended in the context of generalized statistical mechanics in order to control the weighting performed by the Laplace distribution’s tails [15]. However, misfit functions based on Laplace distributions suffer from a singularity issue whenever the residual data is very close to zero [12].

In an attempt to obtain robust and non-singular misfit functions, the geophysical data inversion process has been formulated in the context of generalization of Gauss’ law of error. For instance, Ref. [16] formulated the FWI in the context of Tsallis statistics (also known as *q*-statistics) based on the *q*-generalization of Gauss’ error law [17]. In this regard, the classical and Cauchy distribution based misfit functions are particular cases in the *q* → 1 and *q* = 2 limits, respectively. Indeed, generalizations of Gauss’ law of error based on the foundations of statistical physics have been successfully applied to perform robust physical parameters’ estimation in non-linear geophysical problems, such as misfit functions based on Student’s t distribution [18–20], deformed Gaussian distributions [21–25], generalized maximum likelihood approaches [26–28], non-parametric methods [29], normalized-based objective functions [30, 31], Wasserstein metric from the optimal transport distance [32–34], matching filter techniques [35], as well as in the Rényi framework [36].

In this work, we formulate the FWI based on the generalization of Gauss’ error law linked to the Rényi entropy (or *α*-entropy). The Rényi *α*-entropy [37, 38] was proposed in the context of information theory as a generalization of the Boltzmann—Gibbs—Shannon (BGS) entropy [39], which is very useful to modeling and describing several complex systems in ecology [40, 41], machine learning [42, 43], as well as in quantum entanglement [44, 45]. By considering that the errors are independent and identically distributed by a *α*-generalized Gaussian distribution, which arises from the maximization of the *α*-entropy, we place a robust misfit function in the broad context of the FWI based on the Gauss’ law of error in the Rényi framework. This allows us to perform estimates of physical parameters in an unbiased way.

In addition to mitigating the effects of non-Gaussian errors, another very important issue of FWI is the high computational cost. From a computational point of view, the FWI applications in the mapping of subsurface models with a large extension is limited due to the computational cost of the procedure for solving the wave equation, which is performed several times during the data inversion process. Indeed, target-oriented techniques are crucial to reducing the computational cost of high-resolution seismic images of target areas [46–48]. In this way, to avoid solving the wave equation in the entire physical domain, we propose a new misfit function based on Rényi statistics along with a technique for solving the wave equation only in a target region. Such a methodology inspired from the condensed matter physics named Patched Green’s Function (PGF) [49–51], in which some of us have recently generalized the PGF technique for to apply it in problems of target-oriented modeling [52–55]. The PGF is a powerful methodology to reduce the computational cost in comparison to the classical modeling techniques. This gain in computational time is directly linked to the fact that the PGF computes the wave field just at a target area and receivers positions.

This paper is organized as follows: First, in Section Methodology, after presenting the FWI theoretical foundations in its classical approach, we introduce FWI in the context of Rényi statistics and the PGF technique employing the Lagrangian formalism. Then, in Section Numerical Experiments, we illustrate how our proposal deals with non-Gaussian errors by presenting two numerical examples. In the first one, we consider a Marmousi case study, in which the main goal consists of investigating the robustness of the FWI based on the Rényi *α*-Gaussian distribution regarding erratic data, and, in the second one, we present a realistic application of target-oriented waveform inversion to estimates model parameters in the context of typical Brazilian pre-salt reservoirs. To conclude, in Section Conclusion, we present our final remarks and perspectives.

## Methodology

FWI is a non-linear inverse problem, in which the forward problem consists of modeling the wave propagation through the numerical solution of a wave Eq [1]. In this work, we consider the acoustic approximation that satisfies the following equation:

$$\begin{array}{c} \nabla^{2}\Psi_{s}\left(x,t\right)-\frac{1}{v_{P}^{2}\left(x\right)}\frac{\partial^{2}\Psi_{s}\left(x,t\right)}{\partial t^{2}}=f_{s}\left(t\right)\;\delta (x-x_{s}), \end{array}$$ (1)

where Ψ_*s*_ is the pressure wavefield generated by the seismic source *f*_*s*_, *v*_*P*_ is the P-wave velocity model, $x\in R^{2}$ and $t\in R^{+}$ represent, respectively, the spatial coordinates and the time, and *f*_*s*_(*t*)*δ*(**x**−**x**_*s*_) denotes the source term at the position **x** = **x**_*s*_. The forward problem can also be solved in the frequency domain. Applying the Fourier transform to Eq (1), we obtain the acoustic wave equation in the frequency domain (also known as Helmholtz equation):

$$\begin{array}{c} \nabla^{2}\psi_{s}\left(x,\omega \right)+\frac{\omega^{2}}{v_{P}^{2}\left(x\right)}\psi_{s}\left(x,\omega \right)=F_{s}\left(\omega \right)\;\delta (x-x_{s}), \end{array}$$ (2)

where *ω* is the angular frequency, *ψ*_*s*_ and *F*_*s*_ are the Fourier transform of Ψ_*s*_ and *f*_*s*_, respectively.

The inverse problem consists of inferring the subsurface physical parameters (in our case, the P-wave velocities of the medium) from indirect observations of seismic waveforms (observed data). In the classical approach, the FWI is formulated as a constrained least-squares optimization task as follows:

$$\begin{array}{c} \underset{m}{\text{min}}\;\frac{1}{2}\sum_{\omega }\sum_{s,r}{\left({ϒ}_{s,r}\psi_{s}\left(\omega \right)-d_{s,r}\left(\omega \right)\right)}^{†}(ϒ\psi_{s}\left(\omega \right)-d_{s,r}\left(\omega \right)), \end{array}$$ (3)

subject to,

$$\begin{array}{c} A\left(m,\omega \right)\psi_{s}\left(\omega \right)=S_{s}\left(\omega \right), \end{array}$$ (4)

where the constraint in the latter equation represents the frequency-domain wave Eq (2) in a compact form with **A**(*m*, *ω*) = ∇^2^ + *mω*^2^ and **S**_*s*_(*ω*) = *F*_*s*_(*ω*)*δ*(**x**−**x**_*s*_), in which $m=m\left(x\right)=1/v_{P}^{2}\left(x\right)$ denotes the model parameters (in this case, the squared slowness) and the operator **A** is known as impedance matrix (or Helmholtz matrix) [56, 57]. It is worth emphasizing that the spatial coordinate (**x**) is implicit in Eqs (3) and (4) and henceforth for a simplified notation. *ϒu*_*s*_ and *d*_*s*_ represent modeled data and observed data, respectively, where *ϒ* is a sampling operator (onto the receiver *r* of the source *s*), *ψ*_*s*_ is the solution of Eq (2), and *d*_*s*,*r*_ is the observed data. The superscript † refers to transpose conjugate.

In the minimization problem (3), quasi-Newton methods are widely employed for finding an informative local minimum. In this framework, model parameters are iteratively updated along a descent direction, which can be expressed as:

$$\begin{array}{c} m_{j+1}=m_{j}-\beta_{j}H_{j}^{-1}\nabla_{m}\phi \left(m_{j}\right),\;\text{with}\;j=0,1,2,...,N_{iter}, \end{array}$$ (5)

where $m=1/v_{P}^{2}\left(x\right)$ is the model parameters, *β*_*j*_ > 0 is a step-length computed in the *j*th iteration being *N*_*iter*_ the maximum number of iterations. In large-scales problems, **H**^−1^ represents the *l*-BFGS approximation of the inverse Hessian matrix calculated from previous gradients of a misfit function, ∇_**m**_ *ϕ*(**m**) [58].

In this way, it is remarkable that the gradient of the misfit function is essential in data inversion via FWI. In the classical approach, the misfit function is given by:

$$\begin{array}{c} \underset{m}{\text{min}}\;\phi_{1}\left(m\right)=\frac{1}{2}\sum_{\omega }\sum_{s,r}{\left(ϒ\psi_{s}\left(m,\omega \right)-d_{s,r}\left(\omega \right)\right)}^{†}(ϒ\psi_{s}\left(m,\omega \right)-d_{s,r}\left(\omega \right)) \end{array}$$ (6)

and the misfit function gradient is:

$$\begin{array}{c} \nabla_{m}\phi_{1}\left(m\right)≔\frac{\partial \phi_{1}\left(m\right)}{\partial m}=\sum_{\omega }\sum_{s,r}ℜ\{{\left(ϒ\frac{\partial \psi_{s}\left(m,\omega \right)}{\partial m}\right)}^{†}(ϒ\psi_{s}\left(m,\omega \right)-d_{s,r}\left(\omega \right))\} \end{array}$$ (7)

where *ψ*_*s*_(*m*, *ω*) is a solution of the wave equation. However, note that at each iteration of the FWI, the derivative of the modeled data in relation to each model parameter must be computed, which is computationally very expensive. For this reason, we now present an efficient way to compute ∇_*m*_ *ϕ*_1_(*m*).

In this way, note that the solution of the problem formulated in (3) can be alternatively computed by minimizing the following augmented Lagrangian functional [59]:

$$\begin{array}{c} L\left(m,\psi ,\lambda \right)=\frac{1}{2}\sum_{\omega }\sum_{s,r}{\left(ϒ\psi_{s}\left(\omega \right)-d_{s,r}\left(\omega \right)\right)}^{†}(ϒ\psi_{s}\left(\omega \right)-d_{s,r}\left(\omega \right))\\ +\sum_{\omega }\sum_{s}{\left\langle \lambda_{s}\left(\omega \right),A\left(m,\omega \right)\psi_{s}\left(\omega \right)-S_{s}\left(\omega \right)\right\rangle }_{x}, \end{array}$$ (8)

in which λ is the Lagrange multiplier and 〈.〉_**x**_ denotes the dot product on spatial coordinates **x**.

The minimization of Eq (8) consists of computing the Lagrangian stationary point. Thus, taking the derivative of $L(m,\psi_{s},\lambda_{s})$ with respect to model parameters, pressure wavefield and the Lagrange multiplier, we have:

$$\begin{array}{c} \frac{\partial L(m,\psi ,\lambda )}{\partial m}=\sum_{\omega }\sum_{s}{\left(\frac{\partial A\left(m,\omega \right)\psi_{s}\left(\omega \right)}{\partial m}\right)}^{†}\lambda_{s}\left(\omega \right), \end{array}$$ (9)

$$\begin{array}{c} \frac{\partial L(m,\psi ,\lambda )}{\partial \psi }=\sum_{\omega }\sum_{s,r}{ϒ}^{†}(ϒ\psi_{s}\left(\omega \right)-d_{s,r}\left(\omega \right))+\sum_{\omega }\sum_{s}A^{†}\left(m,\omega \right)\lambda_{s}\left(\omega \right), \end{array}$$ (10)

and

$$\begin{array}{c} \frac{\partial L(m,\psi ,\lambda )}{\partial \lambda }=\sum_{\omega }\sum_{s}(A\left(m,\omega \right)\lambda_{s}\left(\omega \right)-S_{s}\left(\omega \right)). \end{array}$$ (11)

If $\left(\hat{m},\hat{\psi },\hat{\lambda }\right)$ is at the stationary point of the Lagrangian (8), the constraint in Eq (3) is always satisfied after analyzing the latter Eq (11):

$$\begin{array}{c} \frac{\partial L(m,\psi ,\lambda )}{\partial \lambda }{|}_{\begin{array}{c} m=\hat{m}\\ \psi =\hat{\psi }\\ \lambda =\hat{\lambda } \end{array}}=0\;\Rightarrow \;A\left(\hat{m},\omega \right){\hat{\psi }}_{s}\left(\omega \right)=S_{s}\left(\omega \right), \end{array}$$ (12)

which means the wave equation is solved in each FWI iteration.

Now, evaluating Eq (10) at the stationary point of the Lagrangian (8), we obtain:

$$\begin{array}{c} \frac{\partial L(m,\psi ,\lambda )}{\partial \psi }{|}_{\begin{array}{c} m=\hat{m}\\ \psi =\hat{\psi }\\ \lambda =\hat{\lambda } \end{array}}=0\;\Rightarrow \;A^{†}\left(\hat{m},\omega \right){\hat{\lambda }}_{s}\left(\omega \right)=-\sum_{r}{ϒ}^{†}(ϒ{\hat{\psi }}_{s}\left(\omega \right)-d_{s,r}\left(\omega \right)), \end{array}$$ (13)

which is a wave equation similar to Eq (12), but with wavefield λ_*s*_ and the source term of the form $-\sum_{r}{ϒ}^{†}(ϒ{\hat{\psi }}_{s}\left(\omega \right)-d_{s,r}\left(\omega \right))$. Thus, the misfit function gradient is efficiently computed by solving the wave equation in only two moments (in the forward problem and in obtaining the Lagrange multiplier λ).

In summary, to obtain the gradient of the misfit function, it is enough to compute the modeled data by solving the wave equation and correlating it (see Eq (9)) with the solution λ of the wave equation in Eq (13):

$$\begin{array}{c} \nabla_{m}\phi_{1}\left(m\right)≔\frac{\partial L(m,\psi ,\lambda )}{\partial m}=\sum_{\omega }\sum_{s}\omega^{2}\psi_{s}^{†}\left(m,\omega \right)\lambda_{s}\left(m,\omega \right), \end{array}$$ (14)

with

$$\begin{array}{c} A^{†}\left(m,\omega \right)\lambda_{s}\left(\omega \right)=-\sum_{r}{ϒ}_{s,r}^{†}(ϒ\psi_{s}\left(m,\omega \right)-d_{s,r}\left(\omega \right)). \end{array}$$ (15)

The latter equation is known as adjoint wave equation, in which the so-called adjoint wavefield λ is computed by back propagating the errors (see Ref. [60] for more details).

Although the classical approach is quite popular, it is doomed to fail if the errors are non-Gaussian. Indeed, if there are a handful of spurious measurements (outliers) in the dataset, the classical approach estimates biased parameters [13, 61]. It is possible to see such behavior through the analysis of the adjoint-source (right-hand term in Eq (15)). In fact, if there is an outlier into the observed data (*d*_*s*,*r*_ → ∞), we notice that the adjoint wavefield diverges (λ_*s*_ → ∞) since infinite energy is inserted into the reverse wave propagation, and therefore ∇_*m*_ *ϕ*_1_(*m*)→∞.

### FWI based on Rényi *α*-Gaussian distribution

The Rényi entropy (or *α*-entropy) [37, 38] is a one-parameter generalization of the classic BGS entropy, which is useful in information theory. For a continuous random variable *X* with a probability density function *p*(*x*), the *α*-entropy is defined as:

$$\begin{array}{c} H_{\alpha }[p(x)]=\frac{1}{1-\alpha }\text{ln}(\int p^{\alpha }\left(x\right)\;dx), \end{array}$$ (16)

where *α* > 0 and *α* ≠ 1. It is worth emphasizing that the BGS entropy is recovered at the limit *α* → 1:

$$\begin{array}{c} \underset{\alpha \to 1}{\text{lim}}H_{\alpha }\;\overset{\Delta }{=}\;\underset{\alpha \to 1}{\text{lim}}\frac{-\int p^{\alpha }\left(x\right)\;\text{ln}\;(p(x))dx}{\int p^{\alpha }\left(x\right)\;dx}=-\int p\left(x\right)\;\text{ln}\;(p(x))dx=S_{BGS}, \end{array}$$ (17)

in which we have employed the L’Hôpital rule ($\overset{\Delta }{=}$).

From the maximum entropy principle (MEP) for the Rényi *α*-entropy, several statistical distributions have emerged to model and describe a wide variety of complex systems, such as power-law decay in Hamiltonian systems [62] and statistical inference [63]. In this work, we consider an optimal probability function which is derived from the maximization of the *α*-entropy subject to the normalization condition:

$$\begin{array}{c} \int p(x)dx=1, \end{array}$$ (18)

and the unity variance

$$\begin{array}{c} \int x^{2}p\left(x\right)dx=1. \end{array}$$ (19)

In this regard, the *α*-generalized Gaussian probability distribution (or *α*-Gaussian distribution) is a distribution function resulting from the MEP for the Rényi *α*-entropy (Eq (16)) subject to normalization condition (Eq (18)) and the unity variance (Eq (19)) [64–66], which is given by:

$$\begin{array}{c} p_{\alpha }\left(x\right)=Z_{\alpha }{\left[1-(\frac{\alpha -1}{3\alpha -1})x^{2}\right]}_{+}^{\frac{1}{\alpha -1}}, \end{array}$$ (20)

where [*y*]_+_ = max{0, *y*} and *Z*_*α*_ is the normalizing constant given by [65]:

$$\begin{array}{c} Z_{\alpha }=\sqrt{\frac{1-\alpha }{\pi (3\alpha -1)}}\;\frac{\Gamma (\frac{1}{1-\alpha })}{\Gamma (\frac{1+\alpha }{2(1-\alpha )})} \end{array}$$ (21)

for $\frac{1}{3}<\alpha <1$, in which Γ represents the Gamma Function.

By considering that the errors *x* are independent and identically distributed by the *α*-Gaussian distribution (Eq (20)), we obtain the *α*-misfit function using the probabilistic maximum log-likelihood function:

$$\begin{array}{c} \Theta_{\alpha }\left(x\right)=\text{ln}(\prod_{i=1}^{N}p_{\alpha }\left(x_{i}\right))=\text{ln}\{\prod_{i=1}^{N}A_{\alpha }{\left[1-(\frac{\alpha -1}{3\alpha -1})x_{i}^{2}\right]}_{+}^{\frac{1}{\alpha -1}}\}, \end{array}$$ (22)

which can be written as:

$$\begin{array}{c} \Theta_{\alpha }\left(x\right)-N\;\text{ln}\left[A_{\alpha }\right]=\frac{1}{\alpha -1}\sum_{i=1}^{N}\;\text{ln}{\left[1-(\frac{\alpha -1}{3\alpha -1})x_{i}^{2}\right]}_{+}, \end{array}$$ (23)

in which *x* = {*x*_1_, *x*_2_, *x*_3_, ⋅⋅⋅, *x*_*N*_}. We notice that maximizing the latter Eq (23) is equivalent to minimizing the following function:

$$\begin{array}{c} \phi_{\alpha }\left(x\right)=\frac{1}{1-\alpha }\sum_{i=1}^{N}\;\text{ln}{\left[1-(\frac{\alpha -1}{3\alpha -1})x_{i}^{2}\right]}_{+}. \end{array}$$ (24)

In this way, the FWI based on *α*-Gaussian distribution (hereafter *α*-FWI) is formulated as the following minimization task:

$$\begin{array}{c} \underset{m}{\text{min}}\;\frac{1}{1-\alpha }\sum_{\omega }\sum_{s,r}\text{ln}{\left[1-(\frac{\alpha -1}{3\alpha -1}){\left(ϒ\psi_{s}\left(\omega \right)-d_{s,r}\left(\omega \right)\right)}^{†}(ϒ\psi_{s}\left(\omega \right)-d_{s,r}\left(\omega \right))\right]}_{+} \end{array}$$ (25)

subject to the wave equation, **A**(*m*, *ω*)*ψ*_*s*_(*ω*) = **S**_*s*_(*ω*). Thus, the solution of the optimization problem formulated in (25) can be done by minimizing the following augmented Lagrangian *α*-functional:

$$\begin{array}{ccc} L_{\alpha }\left(m,\psi ,\Lambda \right) & =\frac{1}{1-\alpha }\sum_{\omega }\sum_{s,r}\text{ln}{\left[1-(\frac{\alpha -1}{3\alpha -1}){\left(ϒ\psi_{s}\left(\omega \right)-d_{s,r}\left(\omega \right)\right)}^{†}(ϒ\psi_{s}\left(\omega \right)-d_{s,r}\left(\omega \right))\right]}_{+}\\ & +\sum_{\omega }\sum_{s}{\left\langle \Lambda_{s}\left(\omega \right),A\left(m,\omega \right)\psi_{s}\left(\omega \right)-S_{s}\left(\omega \right)\right\rangle }_{x}, & \end{array}$$ (26)

where Λ is a Lagrangian multiplier.

Computing the stationary point of Eq (26), we have:

$$\begin{array}{c} \frac{\partial L_{\alpha }\left(m,\psi ,\Lambda \right)}{\partial m}=\sum_{\omega }\sum_{s}{\left(\frac{\partial A\left(m,\omega \right)\psi_{s}\left(\omega \right)}{\partial m}\right)}^{†}\Lambda_{s}\left(\omega \right), \end{array}$$ (27)

$$\begin{array}{c} \frac{\partial L_{\alpha }\left(m,\psi ,\Lambda \right)}{\partial \psi }=\sum_{\omega }\sum_{s,r}\frac{2{ϒ}^{†}(ϒ\psi_{s}\left(\omega \right)-d_{s,r}\left(\omega \right))}{3\alpha -1-\left(\alpha -1\right){\left(ϒ\psi_{s}\left(\omega \right)-d_{s,r}\left(\omega \right)\right)}^{†}(ϒ\psi_{s}\left(\omega \right)-d_{s,r}\left(\omega \right))}\\ +\sum_{\omega }\sum_{s}A^{†}\left(m,\omega \right)\Lambda_{s}\left(\omega \right), \end{array}$$ (28)

and

$$\begin{array}{c} \frac{\partial L_{\alpha }\left(m,\psi ,\Lambda \right)}{\partial \Lambda }=\sum_{\omega }\sum_{s}(A\left(m,\omega \right)\Lambda_{s}\left(\omega \right)-S_{s}\left(\omega \right)). \end{array}$$ (29)

We notice that Eqs (27) and (29) are exactly Eqs (9) and (11), respectively. This result is expected, since the wave equation constraint is intrinsic to FWI. However, we notice that Eq (28) associated with the pressure wavefield is quite different from the one resulting from the classical approach (Eq (10)). In this way, if $\left(\hat{m},\hat{\psi },\hat{\Lambda }\right)$ is at the stationary point of the Lagrangian (26), we obtain:

$$\begin{array}{c} \frac{\partial L_{\alpha }\left(m,\psi ,\Lambda \right)}{\partial \psi }{|}_{\begin{array}{c} m=\hat{m}\\ \psi =\hat{\psi }\\ \Lambda =\hat{\Lambda } \end{array}}=0, \end{array}$$ (30)

which yields the following *α*-adjoint equation,

$$\begin{array}{c} A^{†}\left(\hat{m},\omega \right){\hat{\Lambda }}_{s}\left(\omega \right)=-\sum_{r}\frac{2{ϒ}^{†}(ϒ\psi_{s}\left(\omega \right)-d_{s,r}\left(\omega \right))}{3\alpha -1-\left(\alpha -1\right){\left(ϒ\psi_{s}\left(\omega \right)-d_{s,r}\left(\omega \right)\right)}^{†}(ϒ\psi_{s}\left(\omega \right)-d_{s,r}\left(\omega \right))}. \end{array}$$ (31)

Note that Eq (31) becomes Eq (13) in the limit *α* → 1.

Thus, the *α*-misfit function is given by:

$$\begin{array}{c} \phi_{\alpha }\left(m\right)=\frac{1}{1-\alpha }\sum_{\omega }\sum_{s,r}\text{ln}{\left[1-(\frac{\alpha -1}{3\alpha -1}){\left(ϒ\psi_{s}\left(m,\omega \right)-d_{s,r}\left(\omega \right)\right)}^{†}(ϒ\psi_{s}\left(m,\omega \right)-d_{s,r}\left(\omega \right))\right]}_{+}, \end{array}$$ (32)

valid for $\frac{1}{3}<\alpha <1$, in which the gradient of the *α*-misfit function is computed by solving the wave equation and correlating it with the solution Λ of the wave equation in Eq (31):

$$\begin{array}{c} \nabla_{m}\phi_{\alpha }\left(m\right)≔\frac{\partial L_{\alpha }\left(m,\psi ,\Lambda \right)}{\partial m}=\sum_{\omega }\sum_{s}\omega^{2}\psi_{s}^{†}\left(m,\omega \right)\Lambda_{s}\left(m,\omega \right), \end{array}$$ (33)

with

$$\begin{array}{c} A^{†}\left(\hat{m},\omega \right){\hat{\Lambda }}_{s}\left(\omega \right)=-\sum_{r}\frac{2{ϒ}^{†}(ϒ\psi_{s}\left(m,\omega \right)-d_{s,r}\left(\omega \right))}{3\alpha -1-\left(\alpha -1\right){\left(ϒ\psi_{s}\left(m,\omega \right)-d_{s,r}\left(\omega \right)\right)}^{†}(ϒ\psi_{s}\left(m,\omega \right)-d_{s,r}\left(\omega \right))}, \end{array}$$ (34)

where the classical misfit function (6) is a particular case in the limit *α* → 1.

In contrast to the classical approach, the *α*-misfit function mitigates the effects of non-Gaussian errors, specially the outliers. It is possible to see such behavior through the analysis of the *α*-adjoint-source (right-hand term in Eq (34)). In fact, if there is an outlier into the observed data (*d*_*s*,*r*_ → ∞), we notice that the adjoint wavefield tends to zero (Λ_*s*_ → 0), and therefore ∇_*m*_ *ϕ*_*α*_(*m*)→0 in that case. Indeed, the *α*-misfit function magnifies small errors and suppresses large ones, since the *α*-adjoint-source (34) is proportional to the errors Λ_*s*_ ∝ Δ*d*_*s*,*r*_ = *Υψ*_*s*_(*m*)−*d*_*s*,*r*_ for small errors and inverse of the errors Δ*d*_*s*,*r*_, Λ_*s*_ ∝ 1/Δ*d*_*s*,*r*_ for large ones. The classical approach (*α* → 1), on the other hand, linearly suppresses small errors and magnifies large errors (λ_*s*_ ∝ Δ*d*_*s*,*r*_), which explain the sensitivity of this approach to non-Gaussian errors [61].

### Target-oriented FWI using PGF techniques

As discussed earlier, solving the wave equation is a critical issue of the FWI technique. In this way, the patched Green’s function (PGF) technique appears as a powerful alternative to reduce the computational cost of imaging problems based on wave equation. In the PGF framework, the solution of the frequency-domain wave equation (see, for instance, the constraint in Eq (3)) is equivalent to solving the following linear system of equations [52, 53]:

$$\begin{array}{c} G^{-1}\psi_{s}=\frac{F_{\omega }}{h^{2}}U_{s}, \end{array}$$ (35)

where the impedance matrix **A** is given by the inverse of the Green’s function **G**^−1^, ***ψ***_*s*_ is the discretized pressure wavefield, and $\frac{F_{\omega }}{h^{2}}U_{s}=F_{s}\left(\omega \right)\;\delta (x-x_{s})$ is the source term in which *h* is the grid spacing (in meters), *F*_*ω*_ is the amplitude of the Fourier coefficients of the seismic source and **U**_*s*_ is a column vector with zero elements everywhere except on the source position **x**_*s*_ representing *δ*(**x**−**x**_*s*_).

It is worth noting that the PGF technique is very useful and powerful since the wave equation solution computational cost is drastically reduced and, consequently, the target-oriented FWI runtime is reduced without losing quality in the seismic data inversion process. In contrast, the conventional FWI formulation is very costly from a computational point of view, since it requires the computation of the wavefield for the entire physical domain.

The PGF method is widely used in condensed matter physics for modeling transport of electronic waves [49, 50] through the calculation of the complete Green’s function **G** of a system using the so-called Dyson equation. By considering that the Green’s function in Eq (35) is invertible, we may apply the Dyson equation to compute **G**^−1^ [67–69]. In this regard, the total Green’s function **G** is associated to a self-energy **g** by employing a connection potential term **V**. We highlight that the connection potential is a very important term in the Dyson equation, as it is responsible for connecting the elements necessary for the calculation of the recursive process, which will be described later. In this way, the impedance matrix **G**^−1^ can be described for the entire computational domain [51, 52]:

$$\begin{array}{c} G=g+\text{gVG}. \end{array}$$ (36)

The idea behind Dyson equation is to make the connection between the main elements of the matrix referring to each Green’s function, in which this connection process is carried out through the connection potential **V**.

In its original formulation, the PGF technique is only valid for wave propagation in a homogeneous medium. However, Ref. [52] reformulated this technique for propagating acoustic waves in disordered (non-homogeneous) media. In this regard, the solution of the wave equation in (35) is efficiently solved by computing the following Green’s functions [53]:

$$\begin{array}{cc} & G_{es}={\left(1-g_{ee}V_{ei}g_{ii}V_{ie}\right)}^{-1}g_{es}, \end{array}$$ (37)

$$\begin{array}{cc} & G_{ts}=g_{ti}V_{ie}G_{es}, \end{array}$$ (38)

$$\begin{array}{cc} & G_{rs}=g_{rs}+g_{re}V_{ei}g_{ii}V_{ie}G_{es}. \end{array}$$ (39)

where the Green’s function is computed just in the target region (represented by index *t*) and at the position of sources (*s*) and receivers (*r*). The subscripts *e* and *i* refer, respectively, to the elements of the outer edge and the inner edge of the target region, connected through the employment of the potential **V**_*i*,*e*_. Eqs (37) and (39) represent the Green’s function computed from the position of sources to receivers. Eq (38) is the Green’s function.

**Fig 1:**
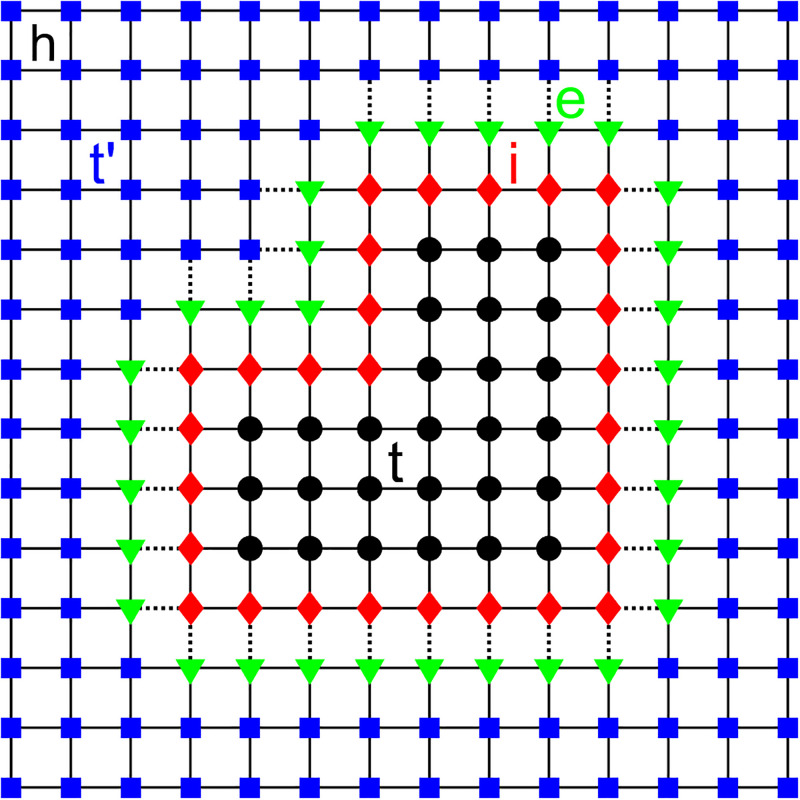
Illustrative scheme of the target and off-target regions, in addition to the connecting elements. The black dots and the blue squares represent, respectively, the target region *t* and the area outside the target region *t*′, in which *h* is the grid spacing. The elements *e* and *i* (represented, respectively, by the downward pointing green triangle and red diamonds) are responsible for the connection between the target region and the outer boundary of the target area.

For a better understanding of how the PGF method works, let us consider a discretized velocity model as shown in Fig 1. In this figure, the black dots represent the target region *t* and the blue squares represent the area outside the target region *t*′, in which *h* is the grid spacing. The PGF method is performed in two steps: First, we find the element that connects the edges of the target area *e* and *i* (see the downward pointing green triangle and red diamonds in Fig 1). In this regard, we calculate the matrices **g**_*rs*_, **g**_*es*_ and **g**_*ee*_ at outside the target area. In the second step called “fill-in”, we compute speed terms on the diagonal of the impedance matrix **G**^−1^ through the connection potential V in all seismic inversion process. Then, we compute the elements of the Green’s function relating the inner edge **g**_*ii*_ of the target region with the points within the target area **g**_*ti*_. The split-in step is performed throughout the inversion process (please see Ref. [54] for more details).

## Numerical experiments

To illustrate how the *α*-PGF-FWI deals with non-Gaussian errors, we present two numerical examples. In the first one, we consider a Marmousi case study, in which the main goal consists of investigating the robustness of the *α*-FWI regarding outliers in the dataset. Then, in the second one, we present an application of the *α*-PGF-FWI to estimates the model parameters in the context of typical Brazilian pre-salt reservoirs. In all numerical experiments, we consider the *limited*-*memory* Broyden-Fletcher-Goldfarb-Shanno (*l*-BFGS) algorithm [70] to solve the minimization problems (Eq (5)), which is a very efficient quasi-Newton method for dealing with large-scale optimization tasks [71]. The *l*-BFGS optimizer fetches an informative (local) minimum of the misfit function using its gradient ∇_*m*_ *ϕ*(*m*). In this way, the *l*-BFGS computes an approximation of the inverse Hessian matrix **H**^−1^ from previous gradients by imposing a secant condition [71]. Moreover, we compute the step-length *β* along the descent-direction search of the gradient, which satisfies the Wolfe conditions [72]. In addition, we consider a Ricker wavelet as the seismic source [73], which is defined as: $f_{s}\left(t\right)=\left(1-2\pi^{2}\mu_{p}^{2}t^{2}\right)\text{exp}\left(-\pi^{2}\mu_{p}^{2}t^{2}\right)$, where *μ*_*p*_ is the peak frequency.

To avoid the inversion crime in all numerical experiments, we generated the observed dataset in the time domain by employing the finite difference method with 2*nd* and 8*th* order approximations for time and space, respectively, in a regular grid with 5*m* spacing with a convolution-perfectly matched layer (C-PML) [74, 75]. In the Marmousi case study, we carried out the FWI in the time domain by employing another forward modeling algorithm using the finite difference method with 2*nd* and 4*th* order approximations for time and space, respectively, in a regular grid with 10*m* spacing with perfectly matched layer (PML) absorbing layers [76] for the spatial discretization. In the Brazilian pre-salt case study, we carried out the FWI in the frequency domain by employing a regular 9-point finite-difference stencil in a regular grid with 25*m* spacing with C-PML absorbing boundaries [76].

### Marmousi case study: Robustness to erratic data

**Fig 2:**
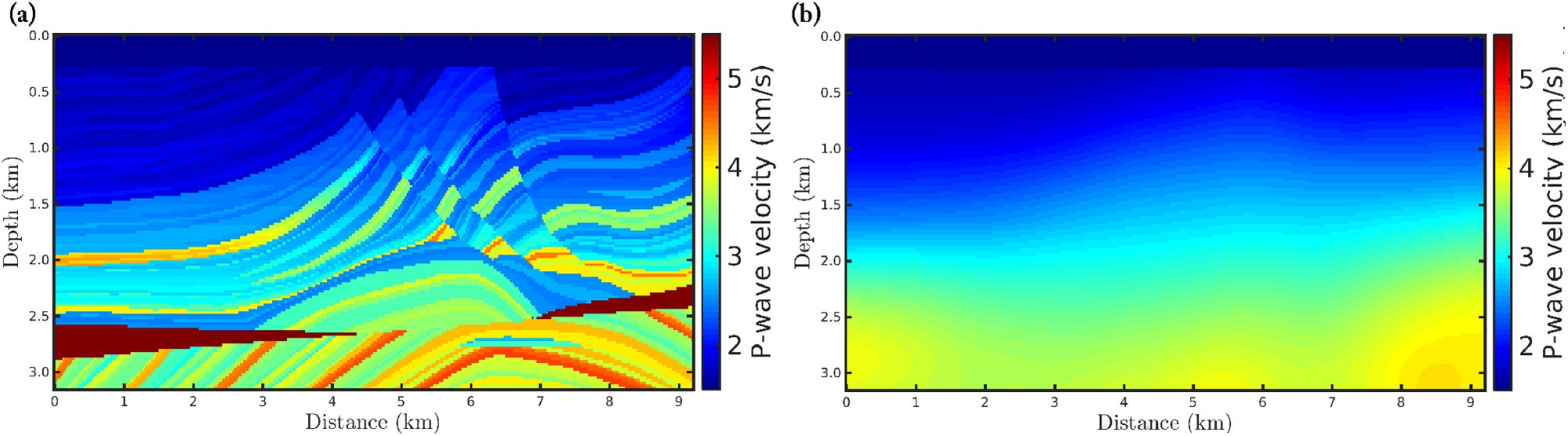
(a) Realistic P-wave velocity model used as the ground truth (true model) representing the Kwanza Basin in Angola, which is known as Marmousi model. (b) Initial model employed in the *α*-FWI tests.

**Fig 3:**
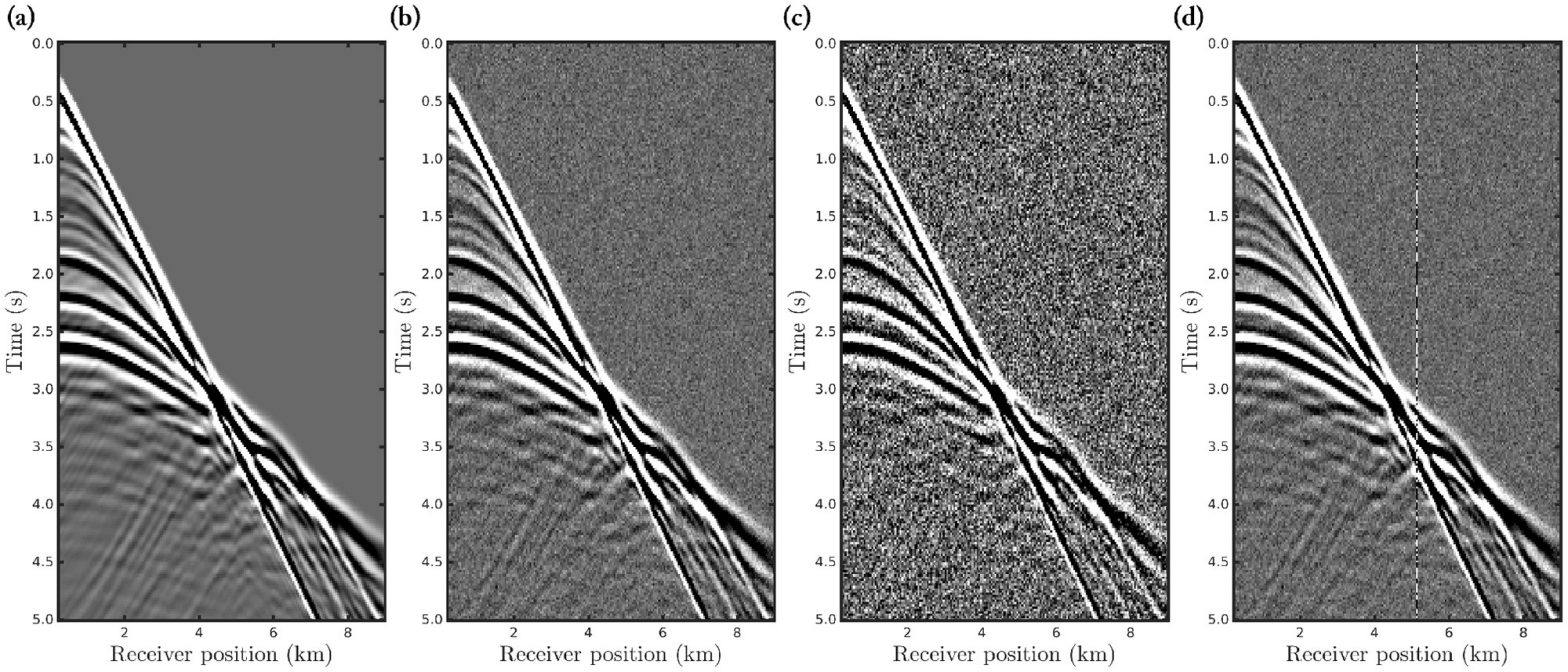
Seismograms (shot-gathers) generated through a seismic survey with the first seismic source at lateral distance 216*m*, for the (a) noiseless data case; the Gaussian noise cases with (b) *SNR* = 70*dB* and (c) *SNR* = 60*dB*; and (d) the Gaussian noise case *SNR* = 70*dB* and erratic data (outliers).

To analyze the robustness of the *α*-FWI regarding erratic data (outliers), we consider a 2D acoustic velocity model which is widely used in geophysical imaging tests, named the Marmousi model. Such a model presents a complex velocity geometry, as depicted in Fig 2(a), and it is based on a realistic region of the Kwanza Basin in Angola [77]. By using the Marmousi model (true model), Fig 2(a), we generate a seismic dataset considering a fixed-spread acquisition at 24*m* in-depth with 183 equally spaced seismic sources spaced 48*m* apart, from 216*m* to 8952*m*, in which we employ a Ricker wavelet with *μ*_*p*_ = 5*Hz* as the seismic source. Furthermore, we consider 23 equally spaced receivers located every 408*m*, from 96*m* to 9072*m*, deployed at the ocean floor (240*m* in-depth) to simulate an ocean bottom nodes (OBN) acquisition, which is a marine type seismic acquisition often used in the last years. The recording time was 5*s*. Fig 3 shows some examples of shot-gathers generated by the first (from left to right) seismic source.

We performed FWI taking into account a noiseless data scenario, to validated our algorithms, and three noisy data scenarios: in the first two, we consider a dataset polluted by Gaussian noise, and then, in the last one, we use a seismic dataset contaminated by spiky-noise (outliers) and Gaussian noise to simulate a realistic circumstance like to Ref. [78]. In the case of the first two scenarios, we consider seismic data contaminated by white Gaussian background noise with signal-to-noise ratio (SNR) of 70*dB* and 60*dB*, respectively, as depicted in Fig 3(b) and 3(c). The SNR is computed by the ratio between the noiseless observed data power and the amplitude noise power. In the fourth scenario, we consider a data set contaminated by Gaussian noise with *SNR* = 70*dB* and that only one of the seismic traces from each receiver is contaminated by outliers (see vertical line in Fig 3(d)). The outliers were added over 5% of the seismic traces by rescaling the amplitude of the signals by a factor of 10*ζ*, in which *ζ* follows a standard Normal distribution. We choose the seismic traces using a uniform distribution.

For each noisy-scenario, we carried out eight data inversions, in which the first one refers to the classical approach (*α* → 1), and the last seven ones are based on the *α*-misfit function with *α* = 0.35, 0.45, 0.55, 0.65, 0.75, 0.85, and 0.95. In all numerical experiments, we inverted for 200 *l*-BFGS iterations from the above-described geometry seismic acquisition and the initial model depicted in Fig 2(b).

**Fig 4:**
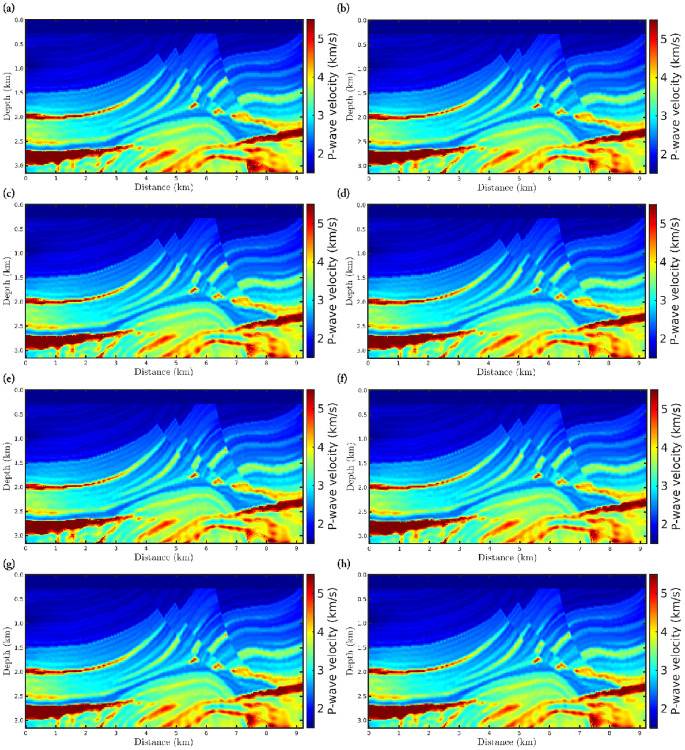
Reconstructed P-wave velocity models in the noiseless data case (first scenario) for the (a) classical FWI (*α* → 1), and *α*-FWI with (b) *α* = 0.95, (c) *α* = 0.85, (d) *α* = 0.75, (e) *α* = 0.65, (f) *α* = 0.55, (g) *α* = 0.45, and (h) *α* = 0.35.

Fig 4 shows the FWI results for the first scenario, in which it is remarkable that the results are satisfactory, since the reconstructed velocity models are close to the true model (Fig 2(a)) regardless of the *α*-value. Such results were already expected once the observed data is not corrupted. We include this scenario just to demonstrate that our algorithms are working fine. To quantitatively compare the reconstructed models with the true model, we consider two statistical measures: (i) the Pearson product-moment correlation coefficient (or Pearson’s *R*, for short); and (ii) the normalized root-mean-square (*NRMS*). The Pearson’s *R* measures the linear correlation between the reconstructed model and the true model, in which it varies between −1 and 1, inclusive. In this regard, *R* = 1 means a strong correlation between the two models and, in our case, *R*-values far from 1 mean low correlation. The NRMS varies from 0 (perfect model) to ∞ (bad model) and is defined as:

$$\begin{array}{c} \text{NRMS}={\left[\frac{\sum_{i}{\left(c_{i}^{true}-c_{i}^{FWI}\right)}^{2}}{\sum_{i}{\left(c_{i}^{true}\right)}^{2}}\right]}^{1/2} \end{array}$$ (40)

where *c*^*true*^ is true model and *c*^*FWI*^ corresponds to the FWI result. The statistical measures for the noiseless data case (first scenario) are summarized in Table 1. Indeed, all reconstructed models are strongly correlated with the true model (*R* ≥ 0.8, according to the strength-scale suggested by Ref. [79]), although the cases *α* = 0.55 and *α* = 0.65 have the highest Pearson’s *R* and the lowest *NRMS* value.

**Table 1:** Statistical measures between the true model and the reconstructed models in the noiseless data case (first scenario). The Pearson’s *R* measures the linear correlations between the models, and the *NRMS* measures the misfit between the true model and the reconstructed models.

Strategy	*α*-value	R	NRMS
classical FWI	*α* → 1.0	0.9129	0.0203
*α*-FWI	*α* = 0.95	0.9164	0.0193
	*α* = 0.85	0.9154	0.0195
	*α* = 0.75	0.9163	0.0193
	*α* = 0.65	0.9176	0.0190
	*α* = 0.55	0.9177	0.0190
	*α* = 0.45	0.9133	0.0201
	*α* = 0.35	0.9124	0.0203

**Fig 5:**
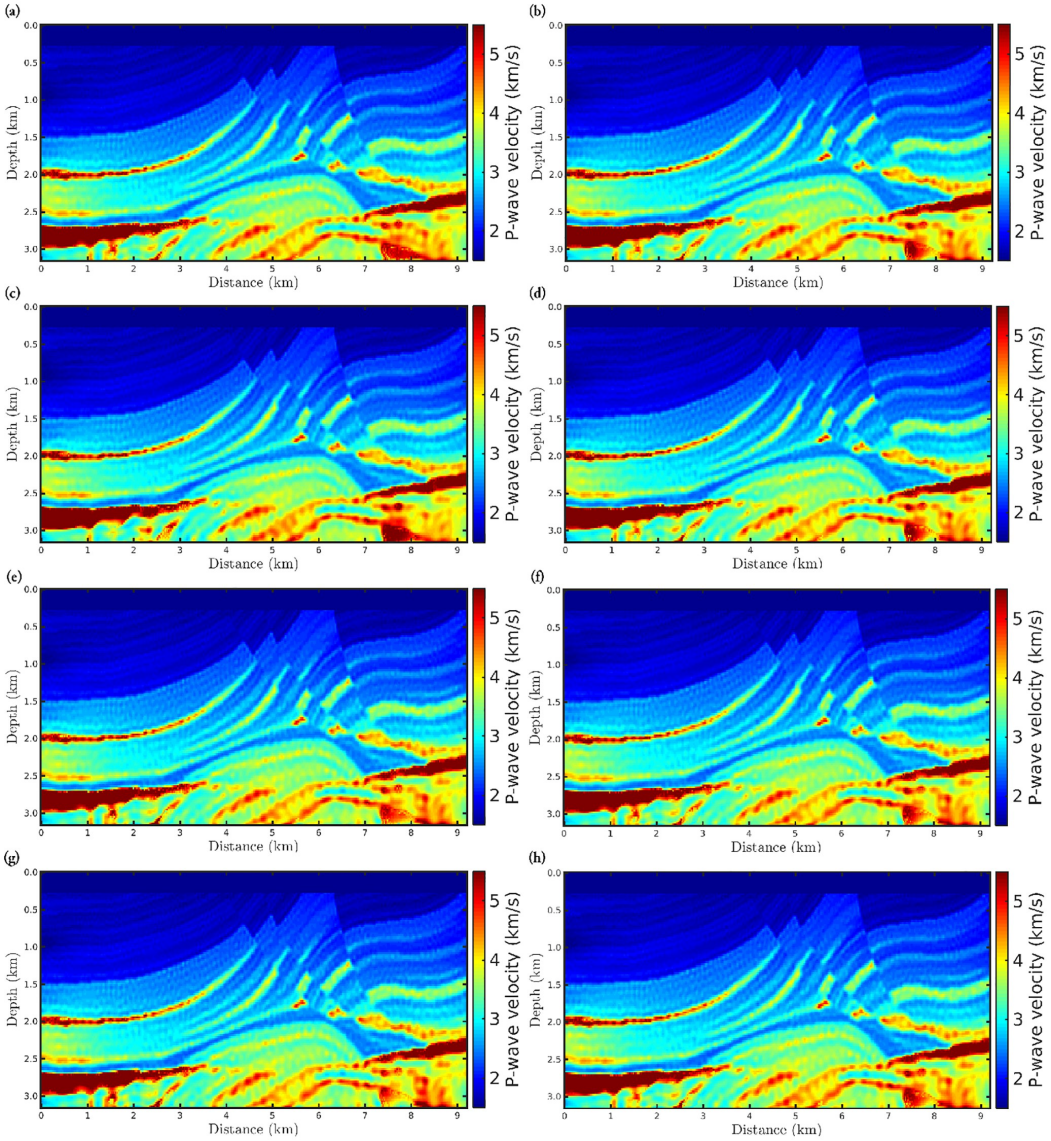
Reconstructed P-wave velocity models in the Gaussian noise case with *SNR* = 70*dB* (second scenario) for the (a) classical FWI (*α* → 1), and *α*-FWI with (b) *α* = 0.95, (c) *α* = 0.85, (d) *α* = 0.75, (e) *α* = 0.65, (f) *α* = 0.55, (g) *α* = 0.45, and (h) *α* = 0.35.

**Fig 6:**
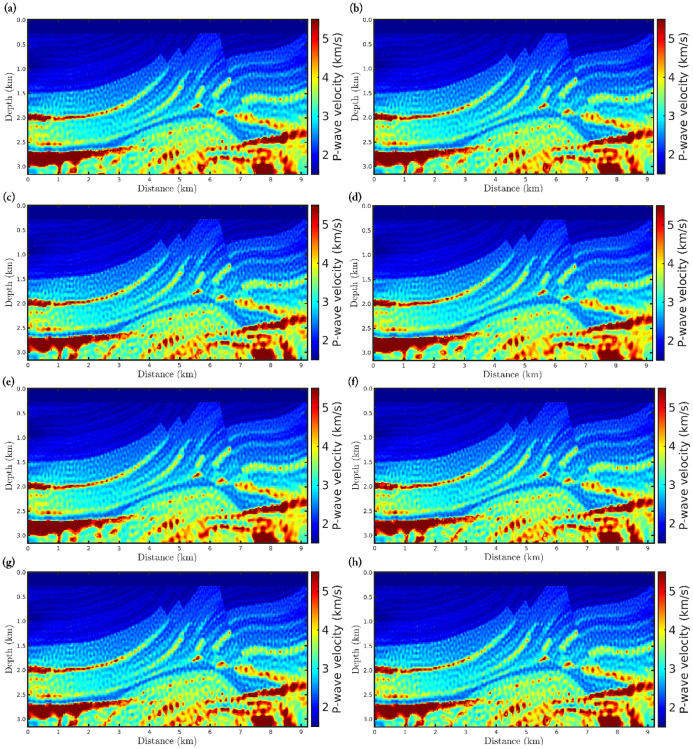
Reconstructed P-wave velocity models in the Gaussian noise case with *SNR* = 60*dB* (third scenario) for the (a) classical FWI (*α* → 1), and *α*-FWI with (b) *α* = 0.95, (c) *α* = 0.85, (d) *α* = 0.75, (e) *α* = 0.65, (f) *α* = 0.55, (g) *α* = 0.45, and (h) *α* = 0.35.

Figs 5 and 6 show the reconstructed models considering the second and third scenarios, respectively, in which the data is contaminated by Gaussian noise. From a visual inspection, we notice that just like the first scenario, the *α*-FWI results are very satisfactory as the resulting models are close to the true model. In fact, regardless of the *α*-value, the reconstructed P-wave models present the main structures of the Marmousi model despite the imprint of the noise. Again, case *α* = 0.55 has a higher correlation and lower *NRMS* error compared to the true model, as summarized in Table 2.

**Table 2:** Statistical measures between the true model and the reconstructed models in the Gaussian noise cases with *SNR* = 70*dB* (second scenario) and *SNR* = 60*dB* (third scenario). The Pearson’s *R* measures the linear correlations between the models, and the *NRMS* measures the misfit between the true model and the reconstructed models.

Strategy	*α*	Second	Scenario (70*dB*)	Third	Scenario (60*dB*)
		R	NRMS	R	NRMS
classical FWI	*α* → 1.0	0.9919	0.0207	0.9692	0.0273
*α*-FWI	*α* = 0.95	0.9907	0.0209	0.9698	0.0272
	*α* = 0.85	0.9835	0.0229	0.9699	0.0271
	*α* = 0.75	0.9906	0.0210	0.9681	0.0275
	*α* = 0.65	0.9920	0.0206	0.9693	0.0271
	*α* = 0.55	0.9922	0.0205	0.9701	0.0270
	*α* = 0.45	0.9936	0.0202	0.9698	0.0271
	*α* = 0.35	0.9911	0.0208	0.9691	0.0275

**Fig 7:**
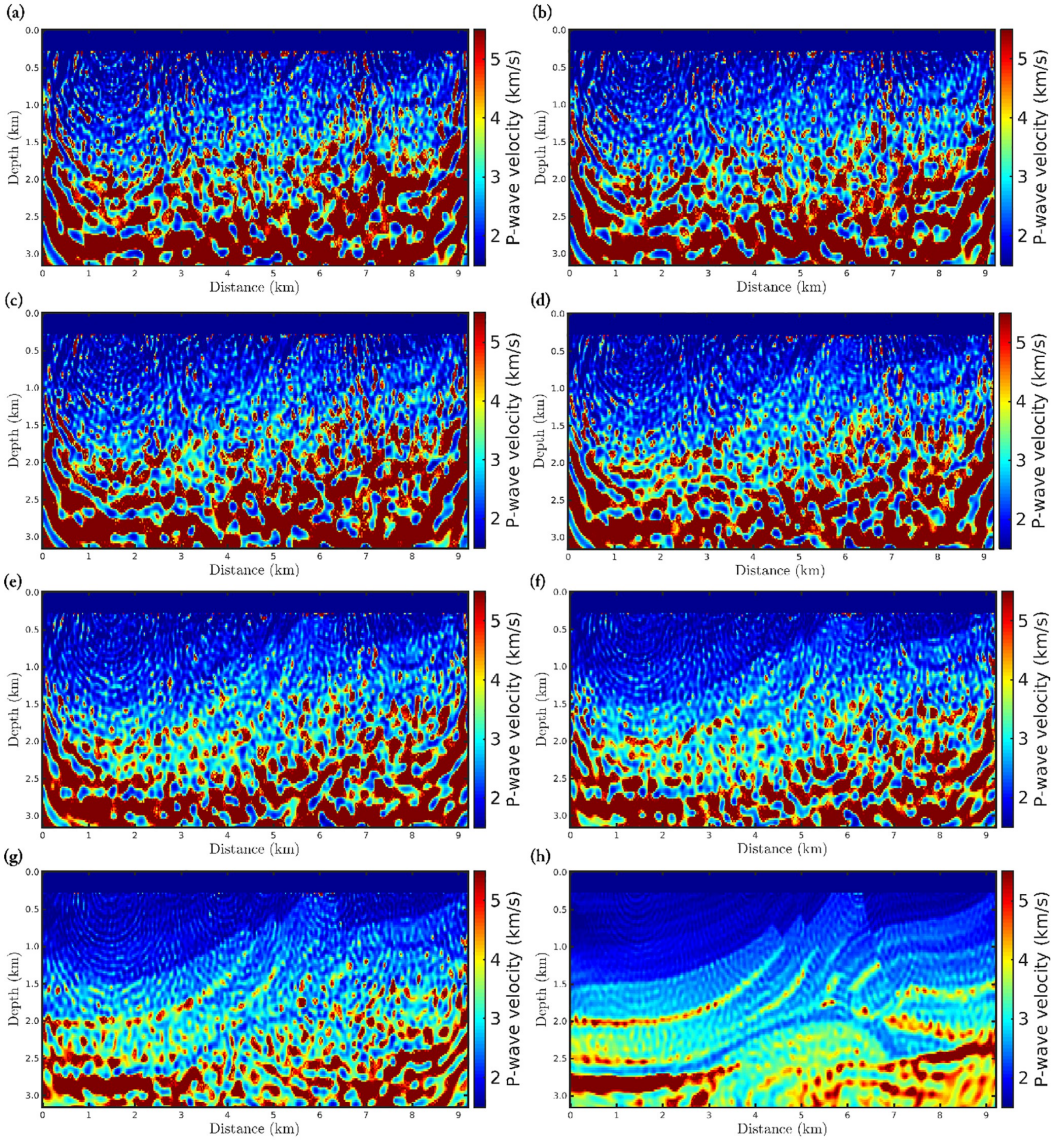
Reconstructed P-wave velocity models in the Gaussian noise case with *SNR* = 70*dB* and outliers (fourth scenario) for the (a) classical FWI (*α* → 1), and *α*-FWI with (b) *α* = 0.95, (c) *α* = 0.85, (d) *α* = 0.75, (e) *α* = 0.65, (f) *α* = 0.55, (g) *α* = 0.45, and (h) *α* = 0.35.

In the fourth scenario, in which non-Gaussian noise is considered, the classical approach (*α* → 1) completely fails to reconstruct a P-wave velocity model, as depicted in Fig 7(a). Such failure of the classical approach is associated with the assumption that the errors are Gaussian, when in practice this is not always true. We notice that as the *α*-value decreases, which means a greater deviation from the Gaussian statistics, the resulting model is more suitable (see Fig 7), especially in the case *α* = 0.35 where the P-wave model is comparable with the reconstructed models in the previous scenarios. In fact, only the *α*-FWI with *α* = 0.35 is strongly correlated with the true model, in addition to having a low error which is in the same order as in the previous scenarios (see Table 3).

**Table 3:** Statistical measures between the true model and the reconstructed models in the Gaussian noise case with *SNR* = 70*dB* and outliers (fourth scenario). The Pearson’s *R* measures the linear correlations between the models, and the *NRMS* measures the misfit between the true model and the reconstructed models.

Strategy	*α*	R	NRMS
classical FWI	*α* → 1.0	0.5789	0.2372
*α*-FWI	*α* = 0.95	0.5942	0.2189
	*α* = 0.85	0.6021	0.2136
	*α* = 0.75	0.6360	0.1831
	*α* = 0.65	0.6500	0.1591
	*α* = 0.55	0.6804	0.1328
	*α* = 0.45	0.7343	0.0889
	*α* = 0.35	0.8432	0.0380

**Fig 8:**
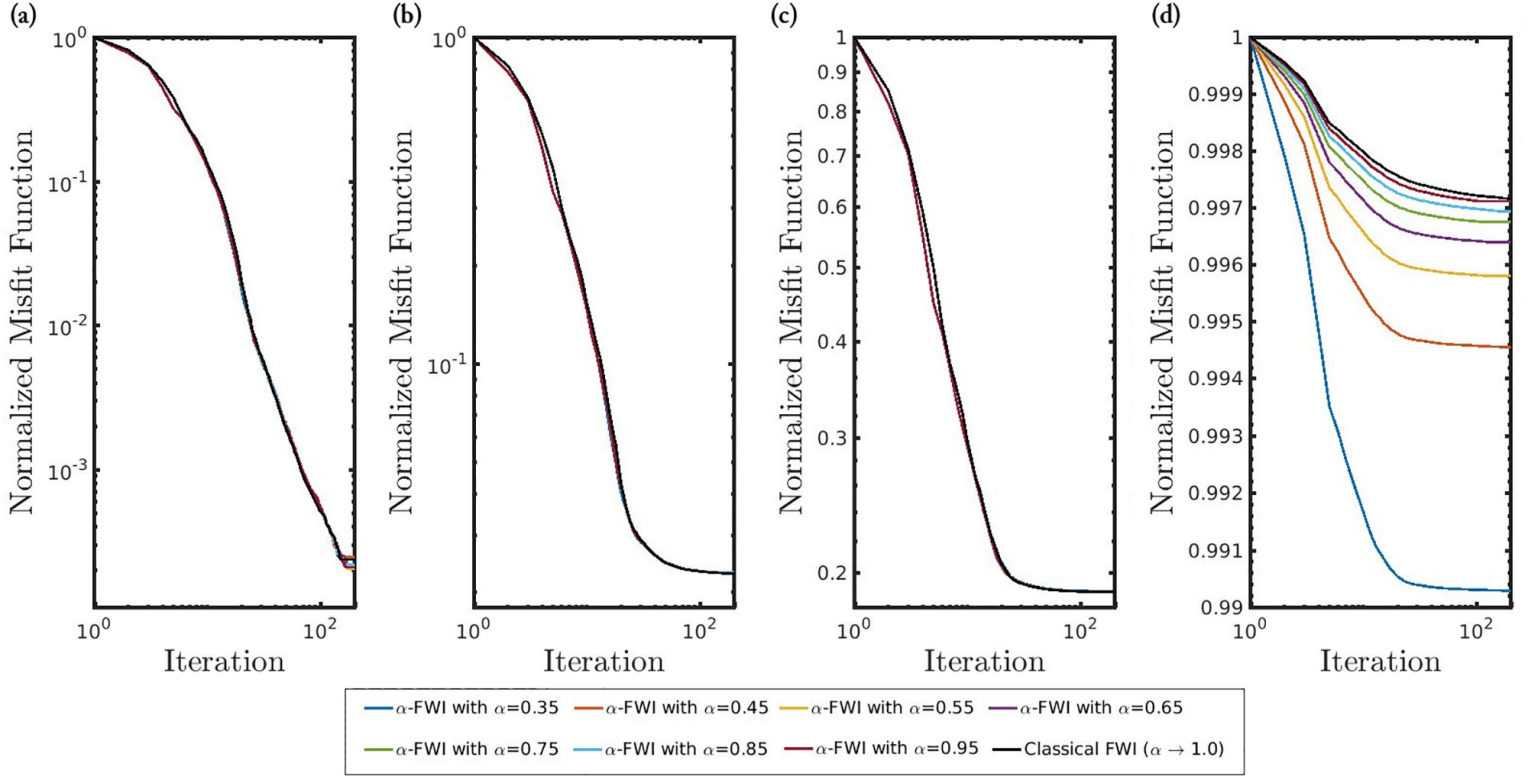
Convergence curves of the *α*-FWI misfit function along the iterations for the (a) noiseless data case; the Gaussian noise cases with (b) *SNR* = 70*dB* and (c) *SNR* = 60*dB*; and (d) the Gaussian noise case *SNR* = 70*dB* and erratic data (outliers).

Fig 8 shows the convergence curves associated with the four scenarios analyzed in this work. We notice that when the seismic data is noiseless (first scenario) or polluted by Gaussian noise (second and third scenarios), the misfit functions decay similarly regardless of the *α*-value, as depicted by the superposition of the black curve over the other ones in Fig 8a–8c. However, when the noises are non-Gaussian (fourth scenario) the classical case rapidly is trapped in a non-informative local minimum (see the black curve in Fig 8d). However, as the *α*-value decreases (increasing deviation from a Gaussian behavior), the misfit function decay is more accentuated (see Fig 8d).

### Brazilian pre-salt case study

**Fig 9:**
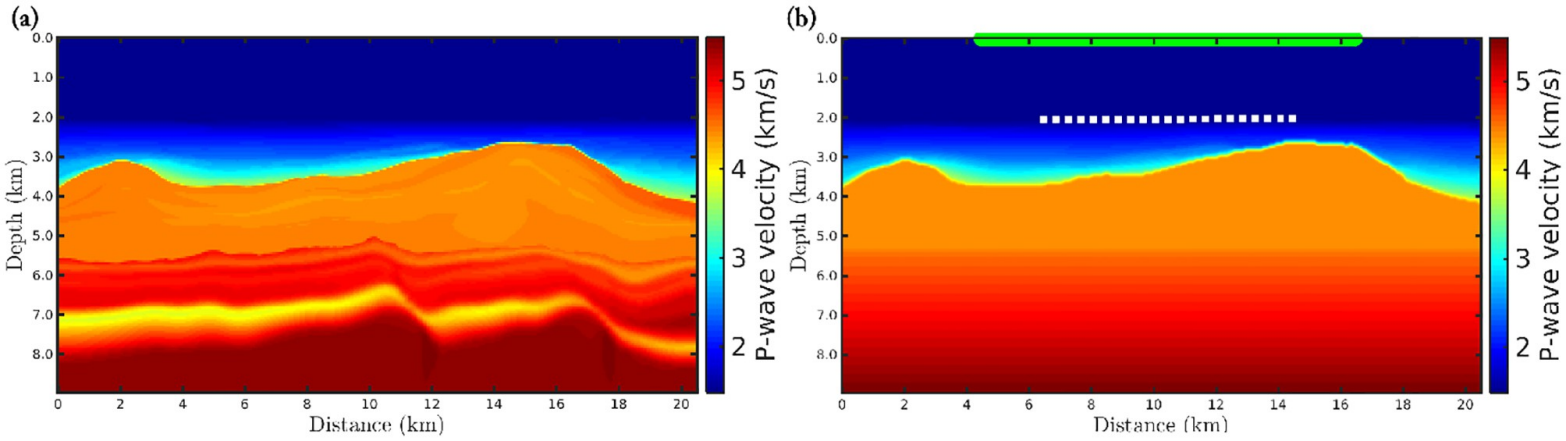
(a) Realistic P-wave velocity model based on the Brazilian pre-salt region, considered as the true model in this study. (b) Typical model of Brazilian pre-salt field modified from [81].

In this section, we explore the potential of the *α*-PGF-FWI for estimating P-wave velocities in a typical Brazilian pre-salt field. In this regard, we consider the realistic acoustic model depicted in Fig 9(a) [80–82] as the true model, which comprises a water layer in which the ocean floor is in average of 2km in-depth, followed by post-salt sediments, a salt body with variable thickness and velocity, the pre-salt reservoir, and the bedrock as the model base. By using the true model, Fig 9(a), we generate a seismic dataset considering an OBN acquisition comprising 21 nodes (receivers) equally spaced every 400*m*, from 6450*m* to 14450*m*, deployed at the ocean floor (see the white squares in Fig 9(b)). In addition, we consider a line of seismic sources, spaced 50m apart, extending by 2km beyond each one end of the node row (see the green line in Fig 9(b)). We call this acquisition geometry OBN classical. The recording time was 10*s*, in which we employ a 5*Hz* Ricker wavelet as the seismic source. We perform several numerical experiments, which will be described next. In this section, in particular, we consider only the *α* → 1 (classical) and *α* = 0.35 cases to compare our proposal with the classical approach. We notice that the water layer in all P-wave velocity models are assumed to be known and, therefore, are kept constant during the inversion process.

The first two experiments consist of imaging the pre-salt region, which remains a great challenge, using the *α*-PGF-FWI. In the first one, we consider the noiseless data set generated by the acquisition geometry described previously. In the second one, we consider the same data set, but polluted by non-Gaussian noise. The non-Gaussian perturbations were generated through a Student’s *t*-distribution with three degrees of freedom. We solve the forward problem by solving the 2-D frequency-domain acoustic wave equation using the finite-difference method, in which we consider a 9-point stencil with a C-PML boundary condition [74, 75] in order to simulate an unlimited medium.

We discretize the Helmholtz operator on a regular grid with a spacing of 25 m. In the inversion process, we consider a multiscale approach [83, 84] in which we employ sequentially three frequency groups: {2, …, 3}, {2, …, 5} and {2, …, 7}Hz. In this framework, the data inversion is performed considering only the content of the data associated with the first group of frequencies, starting from the initial model shown in Fig 9(b). Such a model comprises a water layer, followed by post-salt sediments (which are different from the true model), a salt body with variable thickness and constant velocity (4.4*km*/*s*), and a linear velocity increasing, from 5.4*km* in-depth, rang the second frequencies group. Again, the resulting model is used as the initial model for the third group. For each frequency group, we compute 20 *l*-BFGS iterations.

**Fig 10:**
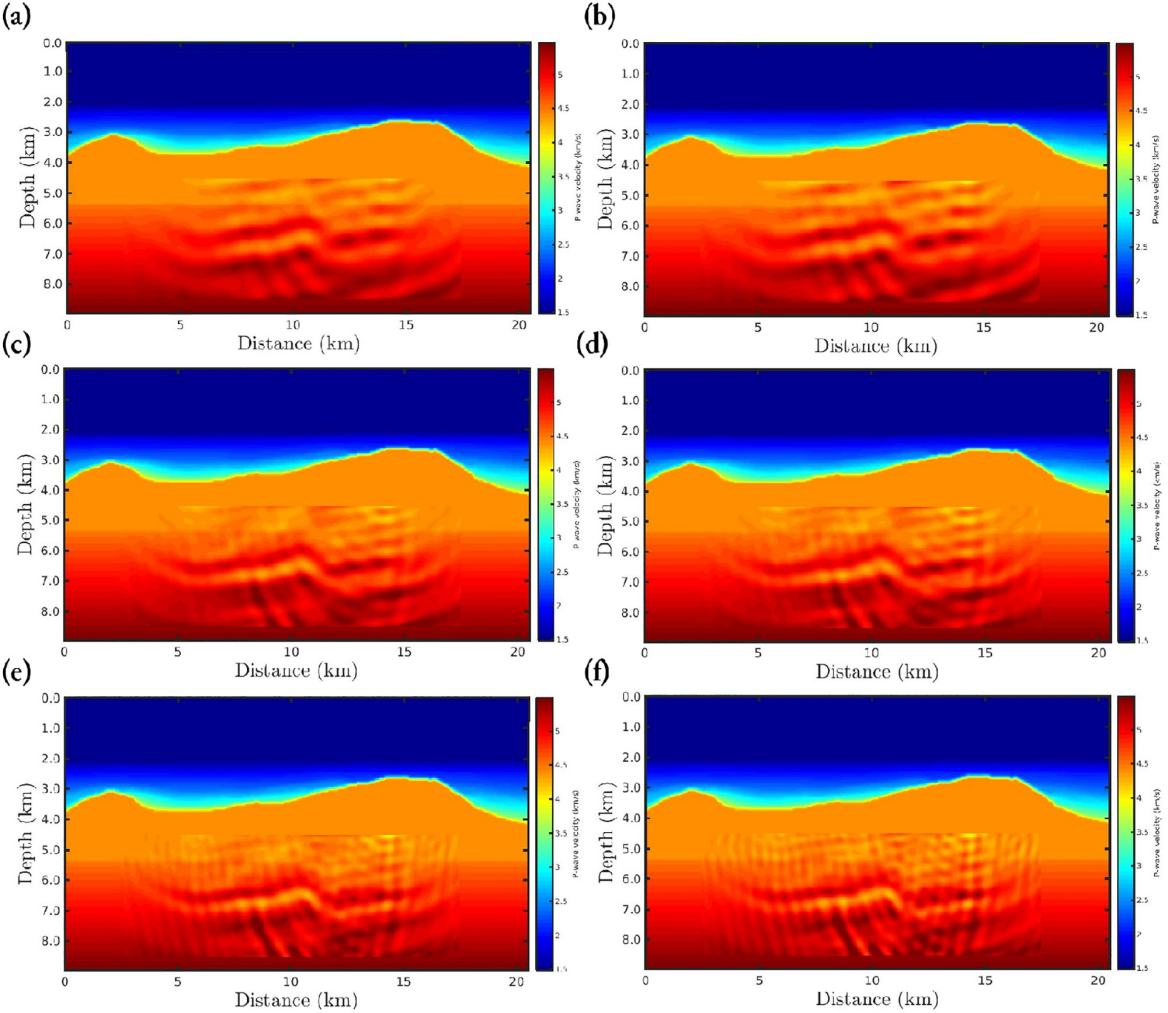
P-wave velocity models reconstructed, for the noiseless case, using *α*-PGF-FWI for the (a)-(b) first, the (c)-(d) second, and the (e)-(f) third frequencies group, in which the left column refers to the classical approach (*α* → 1) and the right column refers to our proposal with *α* = 0.35.

**Fig 11:**
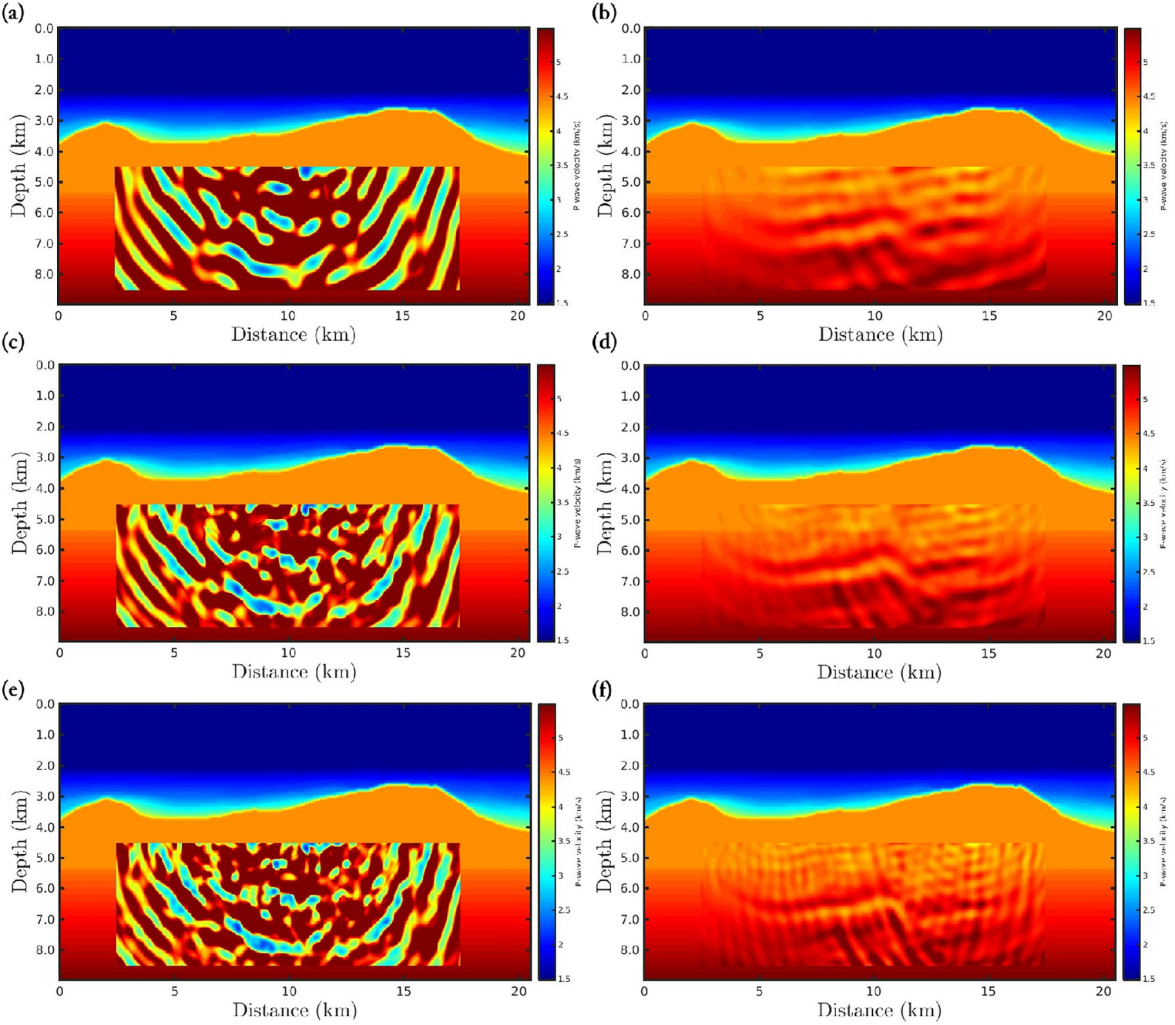
P-wave velocity models reconstructed, for the non-Gaussian noise case, using *α*-PGF-FWI for the (a)-(b) first, the (c)-(d) second, and the (e)-(f) third frequencies group, in which the left column refers to the classical approach (*α* → 1) and the right column refers to our proposal with *α* = 0.35.

The target region consists of a rectangular area, mostly composed of the Brazilian pre-salt, which comprises a region from 4.5*km* to 8.5*km* in-depth and horizontally from 2.5*km* to 17.5*km*, as well-demarcated in Figs 10 and 11. These figures show the *α*-PGF-FWI results for the noiseless and non-Gaussian noise cases, respectively, in which the left column refers to the classical approach (*α* → 1) and the right column refers to our proposal with *α* = 0.35. We notice in Fig 10 that if the data is noise-free, the resulting models are very similar regardless of the applied approach or data-frequency content. On the other hand, when non-Gaussian noise is considered the classical approach fails completely, in which the reconstructed model is biased and therefore very far from the true model. In contrast, our proposal is insensitive to non-Gaussian noise which generates a satisfactory reconstructed model (see the right column of Fig 11) that is comparable to the case where the data is noise-free (Fig 10).

**Fig 12:**
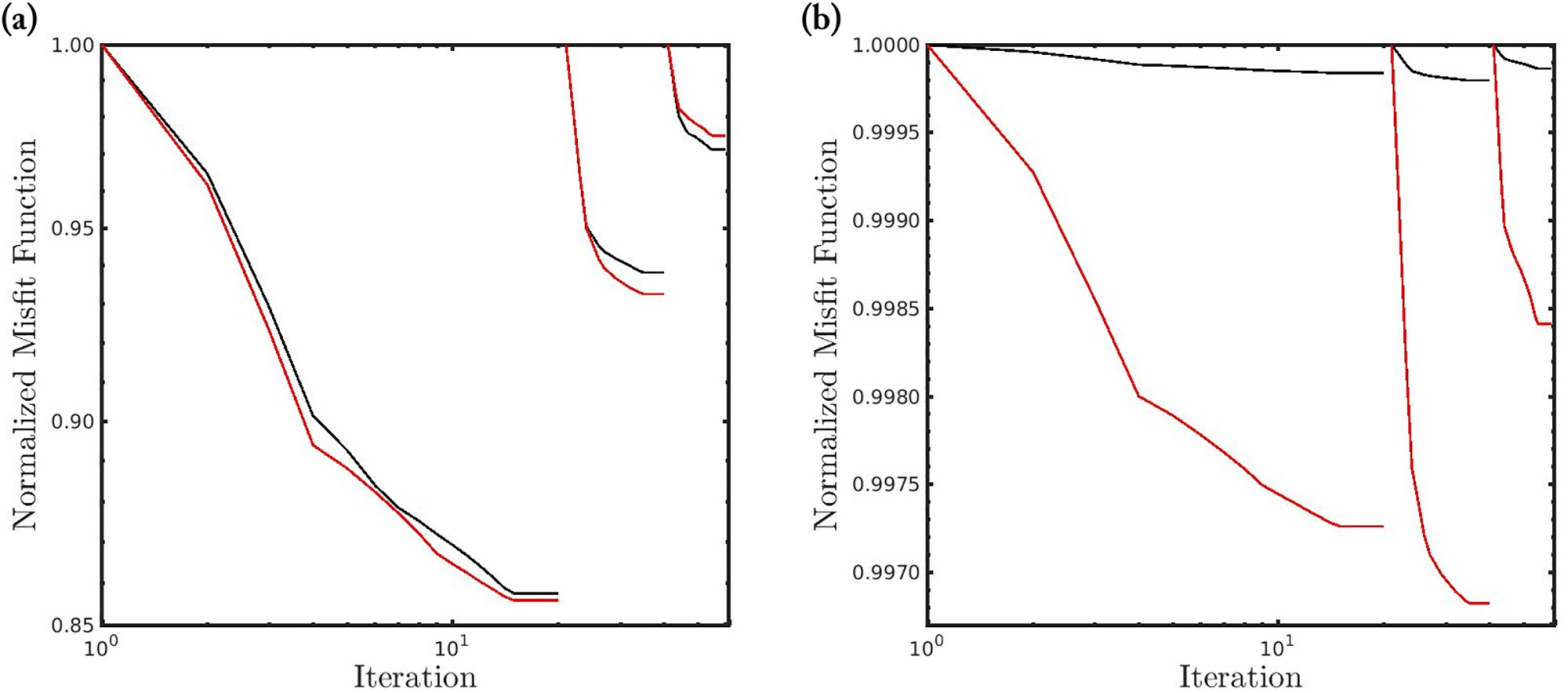
Convergence curves of the *α*-PGF-FWI misfit function along the iterations for the (a) noiseless case, and (b) the non-Gaussian noise case. The black and red curves refer, respectively, to the PGF based on the classical approach (*α* → 1) and our proposed misfit function with *α* = 0.35. The three groupings of curves in each panel refer to the groups of inverted frequencies: {2, …, 3}, {2, …, 5} and {2, …, 7}Hz.

Fig 12 shows the convergence curves associated with the two scenarios analyzed in this case study. We notice that when the seismic data is noiseless (first scenario), the misfit functions decay similarly regardless of the frequency group, as depicted in Fig 12a. However, when the noise is non-Gaussian (second scenario) the classical case rapidly is trapped in a non-informative local minimum similarly to the Marmousi case study (see the black curve in Fig 12a). In contrast, the misfit function of the *α*-PGF-FWI with *α* = 0.35 decays more accentuated (see red curves in Fig 12b).

**Fig 13:**
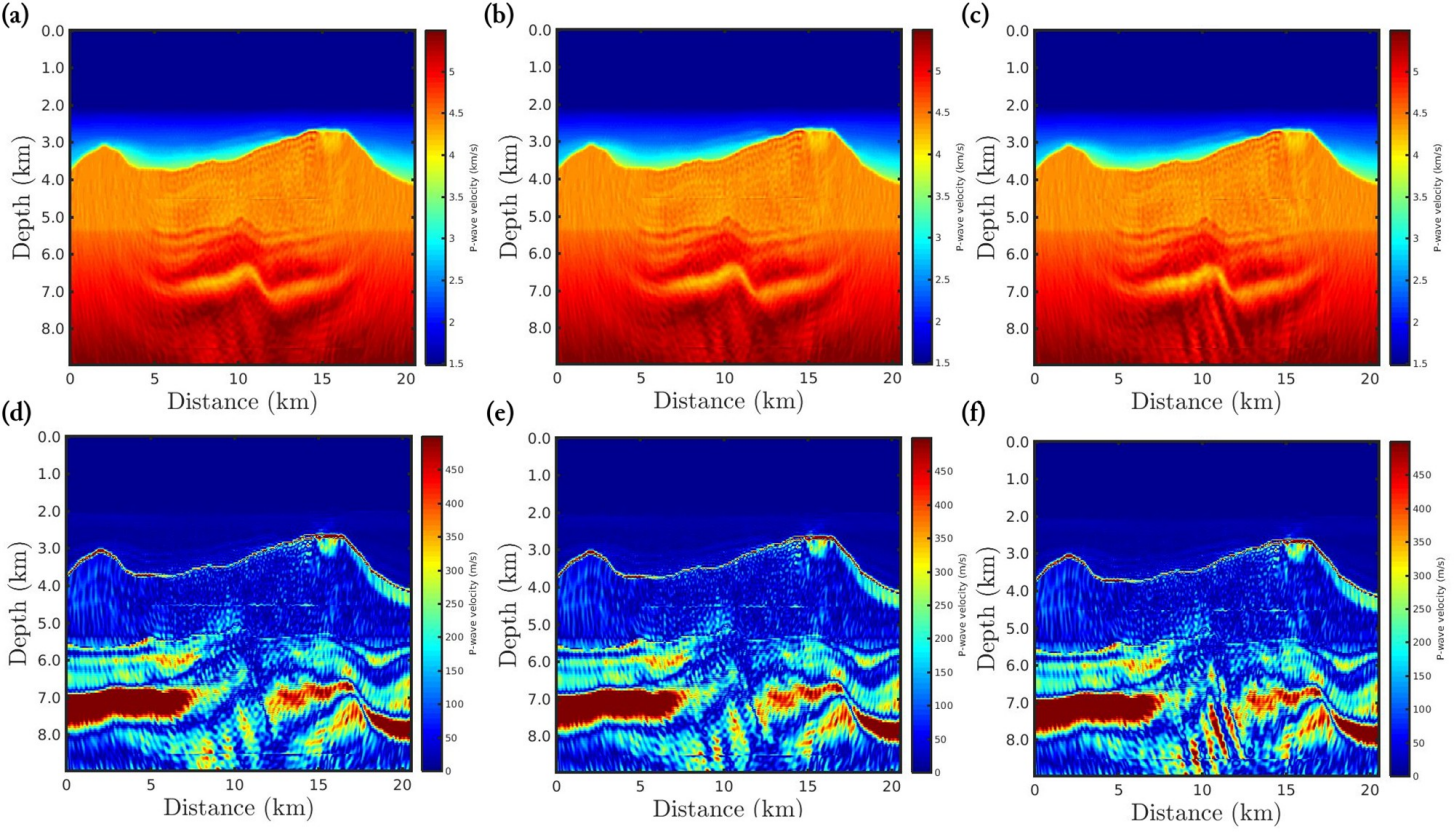
Reconstructed P-wave models from the model in (a) Fig 11(a), (b) Fig 11(d) and 11(f) as initial model, using the time-domain FWI. Panels (d)-(f) show the absolute difference between the reconstructed models shown in panels (a)-(c) and the true model.

Still considering the seismic data contaminated by non-Gaussian noise, we perform three inversions, in time-domain, using *α*-FWI in which the difference between each simulation is just the initial model. In this regard, the P-wave models shown in Fig 11(a), 11(d) and 11(f) are considered as initial model in three different data inversion processes. The resulting models are depicted in Fig 13. In this figure, panels (d)-(f) show the absolute difference between the reconstructed models shown in panels (a)-(c) and the true model. As expected, areas of the P-wave reconstructed models outside the target region have similar results. However, in the pre-salt reservoir region, the initial models presented in Fig 11(a) and 11(d) exhibit less error than the model presented in Fig 11(f), as can be seen by the red fringes in the target region of Fig 13(f).

## Conclusion

To mitigate the effects of non-Gaussian noise and to reduce the computational cost in the reconstruction of P-wave velocity models using the FWI methodology, we proposed a new misfit function based on the Rényi *α*-Gaussian distribution using the PGF method. We call our proposal by the abbreviation *α*-PGF-FWI. The numerical studies with high-resolution complex models demonstrate the effectiveness of our proposal inversion methodology. Using the Marmousi model, we demonstrate the robustness of the Rényi statistics for noiseless circumstances and Gaussian and non-Gaussian noise scenarios. In addition, we have demonstrated that the combination of FWI based on the maximization of Rényi entropy together with the PGF technique is promising for monitoring target regions, such as Brazilian pre-salt reservoirs.

We notice that the *α*-PGF-FWI execution time presented in the Brazilian pre-salt case study is 4.5× less than the conventional frequency-domain FWI (where the entire model is considered). It is worth mentioning that if the observed data does not contain noise or is polluted by Gaussian noise, *α*-PGF-FWI and classical PGF-FWI have similar performances. However, the *α*-PGF-FWI is robust to non-Gaussian noise, while the classical PGF-FWI estimates biased models in these circumstances. The reason is that large errors are better attenuated in the adjoint-source of our proposal compared to the classical approach, leading to a better weighting of waveforms crossing the less illuminated regions of the model. Indeed, the classical misfit function treats all residuals equally, while the proposed misfit function weights residual data according to their amplitude, giving less importance to large errors in the dataset.

The numerical results show that the *α*-PGF-FWI is a powerful methodology to deal with non-Gaussian errors, which may become a valuable tool in geophysical imaging problems, especially when the data is polluted by large errors. It is also worth noting that our proposal can be applied to any problem of estimating physical parameters linked to partial differential equations similar to the Helmholtz equation, such as in biomedical imaging issues [8].

## Data and resources

Plots and numerical simulations were done, respectively, with MATLAB R2016b and Julia Language, on a computer hosting a quad-core (Intel Xeon E5-1620 v3) processor at 3.50GHz and 256GB RAM memories.
